# A Beautiful Saying

**Published:** 1866-08

**Authors:** 


### A BEAUTIFUL SAYING.

"He is happy that finds a true friend in extremity; but he is much more so, who finds not extremity whereby to try his friend."

Many things read well, and, at first glance, strike us as beautifully true; but, on more mature reflection, we cannot but pronounce them to be as false as they are fair. Of these, the quotation above is one, for a higher than mortal authority says: "It is good for me that I have been afflicted." It is ordained that exaltation and humility, alternate joy and sorrow, shall checker as well as fructify the field of Christian life. And as to pecuniary reverses, high authority, as well as a correct observation, show us, "It is good for man that he bear the yoke in his youth."

It is not less true in matters pertaining to human health. We read many things written by so-called "Reformers," which appear "very reasonable;" but their rationality vanishes into thin air, when put to the test of a severe scrutiny. The best advice we can give to our readers is: "Be shy of everything new." Stick to the old paths. But be sure that they are the old ones. The experience of ages is not to be slightly disregarded. We should be slow to abandon what our fathers before us have uniformly found safe and good. If we do change at all, let it not be on the spur of the moment, but only after mature deliberation. The customs of a nation are the practical results of the combined observation of that nation in the course of generations, and, to a considerable extent, are founded on common-sense principles, are the best under the circumstances. Hence every man, however intelligent, should oppose a custom of the country with great diffidence, and not without long and deep investigation. It is for the lack of this, nine-tenths of our "Reform" movements pass to their original nothingness in a very brief space of time. One or two, especially, are *en route* to that destination, which it would not be *proper* now to mention.

